# Associations among Farm, Breed, Lactation Stage and Parity, Gene Polymorphisms and the Fatty Acid Profile of Milk from Holstein, Simmental and Their Crosses

**DOI:** 10.3390/ani11113284

**Published:** 2021-11-17

**Authors:** Eva Samková, Jindřich Čítek, Michaela Brzáková, Oto Hanuš, Libor Večerek, Eva Jozová, Irena Hoštičková, Jan Trávníček, Lucie Hasoňová, Michael Rost, Karolína Hálová, Jiří Špička

**Affiliations:** 1 Department of Food Biotechnologies and Agricultural Products Quality, Faculty of Agriculture, University of South Bohemia in České Budějovice, Studentská 1668, 370 05 České Budějovice, Czech Republic; hasonova@zf.jcu.cz (L.H.); halovk03@zf.jcu.cz (K.H.); 2 Department of Genetics and Agricultural Biotechnology, Faculty of Agriculture, University of South Bohemia in České Budějovice, Studentská 1668, 370 05 České Budějovice, Czech Republic; citek@zf.jcu.cz (J.Č.); vecerek@zf.jcu.cz (L.V.); vondre01@zf.jcu.cz (E.J.); jelini00@zf.jcu.cz (I.H.); rost@zf.jcu.cz (M.R.); 3 Institute of Animal Science, Přátelství 815, 104 00 Praha-Uhříněves, Czech Republic; brzakova.michaela@vuzv.cz; 4 Dairy Research Institute, s.r.o., Ke Dvoru 12a, 160 00 Prague, Czech Republic; hanus.oto@seznam.cz; 5 Department of Animal Science, Faculty of Agriculture, University of South Bohemia in České Budějovice, Studentská 1668, 370 05 České Budějovice, Czech Republic; travnic@zf.jcu.cz; 6 Department of Applied Chemistry, Faculty of Agriculture, University of South Bohemia in České Budějovice, Studentská 1668, 370 05 České Budějovice, Czech Republic; spicka@zf.jcu.cz

**Keywords:** milk fatty acids, genetic and non-genetic factors, *AGPAT6*, *DGAT1*, *LEP*, *FASN*, *SCD1*

## Abstract

**Simple Summary:**

The composition of milk fat has serious importance for consumer health, and unsaturated and especially polyunsaturated fatty acids are preferred. Thus, methods of influencing the fatty acid profile of milk fat are intensively being studied, both genetically and nongenetically. This paper analyzed the effects of polymorphisms in some genes, breeds, lactation parity and stage, and farm on the milk fatty acid profile. The farm was the most significant factor influencing the profile, followed by the lactation stage and their interaction. The lactation parity did not show much importance. The effect of the cow’s breed was minimal. Considering that all 49 individual fatty acids and 11 groups were evaluated, the influence of the gene polymorphisms mentioned was not strong. *SCD1* showed significance in eleven cases.

**Abstract:**

This study aimed to analyze the factors affecting the fatty acid (FA) profile in cow’s milk. The effects of a farm, lactation parity and stage, breed and polymorphisms in the *AGPAT6*, *DGAT1*, *LEP*, *FASN* and *SCD1* genes were evaluated. A total of 196 Holstein cows, 226 Simmental cows and seven crosses were sampled 751 times. The cows were kept at five farms and were in the first up to the sixth lactation, and 49 individual FAs and 11 groups were analyzed. The farm significantly affected the proportion of all FAs except for C16:1n-7*c* and *iso*C14:0. Additionally, the lactation stage was significant for most FAs, and the opposite was true for lactation parity. The effect of the breed was negligible. For the gene polymorphisms, the *SCD1* *TT* genotype exceeded the *CC* in C10:0, C12:0, C14:0, C16:1n-7*c* and C18:2, and the opposite was true for C10:1, C12:1, C14:1n-5*c*, *iso*C17:0, C16:1 and C18:1, i.e., the *TT* genotype was higher for saturated FAs, and the *CT* genotype was higher for monounsaturated FAs. The results hint at the intermediary heredity of the *SCD1* gene. The *FASN* gene was strongly associated with four FAs and branched-chain FAs, and genotype *AG* was better than *GG*. *LEP* was significant for five individual FAs and branched-chain FAs. The differences in FA composition among genotypes were rather small, which could lead to overestimation of the effect and needs to be considered in the next research.

## 1. Introduction

The quality of milk fat is determined mainly by its composition of fatty acids (FAs). Evaluation of factors affecting the FA profile has enormous economic potential. The improvement of milk fat composition can be achieved by non-genetic factors, mainly nutrition, and by breeding.

For the genetic factors, the heritabilities of FA proportions were found to be mostly low [1]. Occasionally, the values were over 0.4, namely, for C6:0, C8:0, C10:0 and C14:0. The heritability of polyunsaturated FAs (PUFAs) was 0.092. Other individual FAs and their groups, saturated FAs (SFAs), unsaturated FAs (UFAs), etc., were of low heritabilities ranging from 0.1 to 0.4. Low to medium heritabilities of FAs ranging from 0.21 to 0.42 was confirmed by Lopez-Villalobos et al. [2]. The data set included measurements in 34,141 milk samples from Holstein-Friesian, Jersey, and their crosses. Similar heritability estimates were found by Park et al. [3] for SFA (0.33) and UFA (0.41) and even higher for MUFA (0.42) and PUFA (0.37). Authors also found that first parity cows had lower heritability for SFAs (0.19) than later parities (0.28). Authors calculated with results of 885,249 repeated test-day milk records. Heritability values may also reflect the origins of FA (performed or synthesized de novo) and their grouping according to saturation or chain length [4].

In the meta-analysis mentioned above [2], all FAs had positive and high genetic correlations with fat and protein percentages of milk, negative and low to medium genetic correlations with milk and protein yields, positive and low to medium genetic correlations with fat yield, and positively low and near to zero correlations with somatic cell score. Except for genetic correlations between group FAs with somatic cell scores, other correlations were significant. A similarly focused study [4] found the same tendencies in genetic correlations. Negative correlations with milk and protein yield and positive correlations with the percentage of fat, protein and casein. The *de novo* synthesized FAs (C6:0, C8:0, C10:0, C12:0 and C14:0) showed strong positive correlations with each other, ranging from 0.24 to 0.99 [2]. SFAs were negatively correlated with UFAs and PUFAs. The SFAs were positively correlated with fat yield and percentage, and the UFAs were negatively associated with both indicators. Such genetic parameters indicate that genetic selection could be used to change the composition of milk fat [2,3]. Moderate correlations between FA and basic indicators of milk performance suggest that the selection program could affect all FA groups; however, not only the desired ones [4].

The effects of dairy (Brown Swiss, Holstein, Jersey) and dual-purpose (Simmental, Alpine Gray) breeds were analyzed [5]. Holstein cows produced more milk than the other cattle breeds, with the highest *trans* FA (TFA) and C18:1 and the lowest C18:0 proportion. Comparisons between dairy and dual-purpose breeds highlighted significant differences for all traits except for PUFA and TFA. Dairy breeds had greater SFAs, short-chain FAs (SCFAs), medium-chain FAs (MCFAs), C14:0 and C16:0 proportions, and dual-purpose breeds produced milk with higher proportions of monounsaturated FAs (MUFAs), long-chain FAs (LCFAs), C18:0 and C18:1. Dairy breeds produced more milk than dual-purpose breeds, but the milk FA profile of the latter was more favorable from a human nutrition point of view.

In addition to genetic parameters, the effects of major genes were studied. The polymorphisms of the *SCD1* (*stearoyl-CoA desaturase 1*) gene significantly affected the milk fat percentage and the proportions of some FAs [6]. The authors found the optimal FA composition of milk in cows of the *TT* genotype. Other association studies revealed that *FASN* (*FA synthase*), *PPARGC1A* (*peroxisome proliferator-activated receptor gamma coactivator 1-alpha*), *ABCG2* (*ATP-binding cassette, sub-family G, member 2*) and *IGF1* (*insulin-like growth factor 1*) gene polymorphisms were mainly associated with MCFAs and LCFAs, especially *FASN* for C10:0, C12:0 and C14:0 [7]. Their findings provide evidence for the possible selection of dairy cows with a healthier milk FA composition by genomic selection schemes. Similarly, other authors have suggested a genomic approach to a complex description of the genetic background; this option helps to increase the chances of identifying loci associated with economically important traits [8,9]. The significant effect of *FASN* gene polymorphisms was also confirmed by other authors [10]. Additionally, other gene polymorphisms were studied in relation to the FA profile, for example, *GH* (*growth hormone*), *MSTN* (*myostatin*), *FADS2* (*fatty acid desaturase 2*), *CPM* (*carboxypeptidase*), *THRSP* (*thyroid hormone-inducible hepatic protein*), *ACACA* (*acetyl-CoA carboxylase alpha*), *AGPAT3* (*1-acylglycerol-3-phosphate O-acyltransferase 3*) [11,12,13,14,15,16,17]. The authors suggest that some of them could be useful as a marker selection for FA composition in milk. Specifically, *DGAT1* (acyl-CoA diacylglycerol transferase 1) and *SCD1* polymorphisms have recently become the focus [6,18,19,20,21].

This paper analyzed the effect of polymorphisms of 1-acylglycerol-3-phosphate O- acyltransferase 6 (*AGPAT6*), leptin (*LEP*), *DGAT1*, *FASN* and *SCD1* genes, breed, farm, lactation parity and stage on the proportion of FAs in cow´s milk. Additionally, the interactions of genotype × farm, genotype × lactation stage, farm × lactation stage and lactation parity × lactation stage were analyzed. So, we aimed to contribute to the knowledge of the potential influence of lipogenic gene polymorphisms on the FA profile in cow milk; this study also comprised other effects to evaluate the polymorphisms under field conditions.

## 2. Materials and Methods

### 2.1. Animals

The group analyzed (n = 429 with 751 measurements in all) consisted of cows of Holstein (n = 196 with 342 measurements) and Simmental (n = 226 with 396 measurements) breeds in the Czech Republic and their crosses (n = 7 with 13 measurements). The cows were kept on five farms in free housing (n_1_ = 132; n_2_ = 125; n_3_ = 77; n_4_ = 49; n_5_ = 46). The milk samples were obtained once or twice from September to December 2015, and the cows were in the first up to the sixth lactation. There were 114 cows with 202 measurements on the day in milk (DIM) 1–99, 157 cows with 296 measurements on DIM 100–200, and 158 cows with 253 measurements on DIM 201–305. The feed ratio consisted of maize silage, grass silage, hay and feed concentrate year-round.

### 2.2. Fatty Acids Analyses

Milk samples were analyzed for their MFAP (the proportion of FAs in milk fat) using the gas chromatography (GC) method after previous lyophilization of the material, fat extraction and derivatization of the FAs at the Department of Applied Chemistry (the University of South Bohemia in České Budějovice, Faculty of Agriculture). The determination of the FAs was performed on a Varian 3800 instrument (Varian Techtron, Palo Alto, CA, USA). The identification of FAs in the milk fat was performed using Supelco standards according to Samková et al. [22]. The FA proportions were stated as g/100 g of FAs.

### 2.3. Genotyping

DNA was isolated from the milk samples using a MagCore HF16 Plus DNA/RNA extractor (RBC Bioscience, New Taipei, Taiwan). Genotyping was performed by the PCR/RFLP method. *DGAT1* gene alleles *A* (alanine) and *K* (lysine) were genotyped according to the methods of Kuhn et al. [23]; *LEP* gene alleles *M* and *W* were genotyped according to Buchanan et al. [24]; *FASN* gene alleles *A* and *G* were genotyped according to Roy et al. [25]; *SCD1* gene alleles *C* and *T* were genotyped according to the methods of Inostroza et al. [21]. *AGPAT6* gene alleles *C* and *T* were genotyped using fragment analysis as in Littlejohn et al. [26]. The sequences of the primers used in the PCR and restriction endonucleases used for genotyping are given in Table S1. Absolute frequencies of samples from cows with respective genotypes are given in Table 1.

### 2.4. Statistical Analysis

Statistical analyses were performed using SAS (SAS 9.4, SAS Institute, Cary, NC, USA) [27]. The data set contained repeated measurements of FA obtained in the first to sixth lactations. A linear mixed model (MIXED procedure of the SAS system with repeated measurements) and the LSM method were used to analyze the effects of the polymorphisms and other effects on the FA proportions. The model was developed as follows:

FA_ijklmnop_ = µ + gene_i_ + farm_j_ + lac_k_ + lacstage_l_ + breed_m_ + sire_n_ + pe_o_ + e_ijklmnop_

where FA_ijklmnop_ = fatty acid (FA group); µ = mean; gene_i_ = fixed effect of the gene (class effect i = 1, 2, 3); farm_j_ = fixed effect (class effect j = 1, …, 5); lac_k_ = fixed effect of the lactation parity (class effect k = 1, …, 6); lacstage_l_ = the fixed effect of the lactation stage (class effect l = 1, 2, 3 for DIM 1–100, 100–200, 200–305); breed_m_ = the fixed effect of the breed (class effect m = 1,2,3); sire_n_ = random effect of the father of the cow; pe_o_ = permanent environment of the cow (repeated measurement); e_ijklmnop_ = random residual effect.

The interactions genotype * farm, genotype * lactation stage, farm * lactation stage and lactation parity * lactation stage were tested by the same model equation by adding the fixed effect of interaction depending on chosen combination as follow:

FA_ijklmnop_ = µ + gene_i_ + farm_j_ + lac_k_ + lacstage_l_ + breed_m_ + (interaction)_xy_+ sire_n_ + pe_o_ + e_ijklmnop_

where FA_ijklmnop_ = fatty acid (FA group); µ = mean; gene_i_ = fixed effect of the gene (class effect i = 1, 2, 3); farm_j_ = fixed effect (class effect j = 1, …, 5); lac_k_ = fixed effect of the lactation parity (class effect k = 1, …, 6); lacstage_l_ = the fixed effect of the lactation stage (class effect l = 1, 2, 3 for DIM 1–100, 100–200, 200–305); breed_m_ = the fixed effect of the breed (class effect m = 1,2,3); interaction_xy_ = interaction between fixed effects were following: (gene * farm)_ij_, (gene * lacstage)_il_, (farm * lacstage)_jl_ or (lac * lacstage)_kl_, only one interaction was always analyzed; sire_n_ = random effect of the father of the cow; pe_o_ = permanent environment effect of the cow (repeated measurement); e_ijklmnop_ = random residual effect.

The Tukey–Kramer test was used for post-hoc comparisons.

## 3. Results and Discussion

The results of studies on relationships between gene polymorphisms and fat content in cow milk made by many authors are at disposal and results between gene polymorphisms and FA profile as well. However, the last-mentioned are less frequent, but some authors suggest incorporating the profile of FAs into the breeding programs [3]. To determine the polymorphism of the *AGPAT6, DGAT1, LEP, FASN* and *SCD1* genes, 49 FAs and 11 FA groups were assessed. Table 2 shows the *p*-values of those with a significant effect of the polymorphous gene, and those with a *p*-value near the significance threshold. Table S2 shows the least squares means ± standard error of all genotypes and all FAs.

For the individual FAs, polymorphisms in the *SCD1*, *LEP* and *FASN* genes showed the highest counts of significance (*p* < 0.05), namely, 11, 5 and 4. Somewhat surprisingly, the *DGAT1* polymorphism did not show such an influence; the *AA* genotype was superior to *AK* only in C22:0 (*p* < 0.05) and *AK* over *AA* in C20:4n-3 (*p* < 0.01). The influence of *DGAT1* gene polymorphisms on the fat content in cow´s milk has been confirmed repeatedly [18,19,28]. Other authors found that polymorphisms strongly influenced MCFAs and UFAs in *DGAT1* and *SCD1*. Other regions also showed significant associations with the FAs studied, and these additional regions explained a relatively small percentage of the total additive genetic variance [29]. In the *DGAT1* gene, cited authors found an association of the *K* allele with higher proportions of C6:0, C8:0, C16:0 and C16:1, and with lower proportions of C14:0, C18:1 and conjugated linoleic acid (CLA), and their findings still suggest that an additional QTL (quantitative trait locus) may be present for the unsaturated MCFAs on BTA 14, where *DGAT1* is located. Moreover, the authors found an association of *SCD1* polymorphism with C10:0, C14:0, C10:1, C12:1, C14:1 and C16:1, whereas C16:0 was not significant. These results for *SCD1* agree quite well with those of ours. In our analysis, the *SCD1 TT* genotype exceeded the *CC* in C10:0, C12:0, C14:0, C16:1n-7*c* and C18:2, and in contrast, the *CC* genotype exceeded the *TT* in C10:1, C12:1, C14:1n-5*c*, *iso*C17:0, C16:1 and C18:1, i.e., the *TT* genotype was better for saturated FAs, and the *CC* genotype was better for MUFAs, as in Inostroza et al. [21]. Additionally, others found that the *SCD1* genotype was important for the FA relative proportion [20,30]. In this paper, the results hint at the intermediary heredity of the *SCD1* gene.

In this paper, the polymorphism in the *FASN* gene showed a strong association with *iso*C14:0, C16:1, C20:1n-9*c* and C22:5n-3; in all cases, the heterozygous genotype *AG* was higher than the *GG* genotype, the same for the BCFA group. In our group of cows, the *AA* genotype was rare, so association analysis was not possible, which may be a consequence of indirect selection for other performances. The abovementioned Bouwman et al. [29] also found this gene to be a candidate gene, mainly for SCFAs and saturated MCFAs and with unsaturated LCFAs. In contrast to our results, Inostroza et al. [21] reported that the *FASN GG* genotype was better for the proportions of C14:0, C16:0, C18:0, C18:1n-9*t*, C18:1n-9*c* and MUFAs.

In the *LEP* gene, the genotype was significant for five individual FAs, both saturated and unsaturated, and the BCFA group, and the three *p-*values were near the significance threshold. In all cases, the *WW* genotype outperformed the *MW* and *MM* genotypes.

In the *AGPAT6* gene, three *p*-values were near the significance threshold. The lowest proportion of FAs was always found for the *CC* genotype. In contrast, Littlejohn et al. [26] reported that the *AGPAT6* polymorphism was significantly associated with the proportions of a range of milk FAs, and the difference may be influenced by incorporating more effects in our analysis. Nafikov et al. [31], accordingly with our results, did not find associations between *AGPAT6* polymorphisms and milk FA composition.

Interestingly, the polymorphisms of the *LEP*, *FASN* and *SCD1* genes did not overlap with the significantly affected FAs. Only the C22:5n-3 and BCFA groups showed significance for both the *LEP* and *FASN* genes; C14:0 and C16:1 for *FASN* and *SCD1* genes.

The variance in fat percentage is attributable to a relatively low number of QTLs [32], namely, four major QTL regions explained 46.18% of the estimated breeding value variance. However, our results hint at a more intricate genetic background for the milk fat composition, but with the low heritabilities mentioned in the chapter Introduction.

The breed of cows hardly ever affected the FA proportion (Table 3, Table 4, Table 5, Table 6 and Table 7). Only the saturated *iso*C14:0, C16:0, *iso*C16:0, C20:0 and C22:0 showed significance, and C14:1n-5*c*, C16:1n-7*c*, C20:4n-3 were practically identical in models with all genes. Maurić et al. [10] found a slightly higher effect of the breed when comparing the Simmental and Holstein Simmental crossbreeds. In another study, weak effects were found [33], as in our analysis.

For the nongenetic factors, the farm significantly affected all FAs with sporadic exceptions, namely, C16:1n-7 and *iso*C14:0 (Table 3, Table 4, Table 5, Table 6 and Table 7). It hints at the crucial impact of feeding on performance and reflects low heritability [1,2]. The effect was significant even though the feeding was generally similar on all farms, i.e., conserved fodder year-round. However, even slight differences in the proportions of components, the composition of grasslands and feed supplements can alter the profile of FAs in milk fat [33,34,35,36]. The results of mentioned studies showed that there are some significant differences between hay milk and silo milk. Linoleic acid, alfa-linolenic acid showed higher concentrations in hay milk. The feeding of grass forage as hay improves the efficiency of forage utilization by cows [37]. Significant effects of the month of the year were identified for milk concentrations of individual FA, FA groups and FA indices [38].

The lactation stage is associated with changes in milk production and the physiological condition of the cow, and it significantly influences the profile of FAs. Some FAs, namely, C8:0, C10:0, C15:1, C16:1n-9, *anteiso*C17:0, C18:2n-6, C18:3, C21:0, C20:4n-6, PUFA and SCFA, did not show significant differences among lactation stages, i.e., their proportion from delivery to the end of lactation was relatively stable. The effect of the lactation stage is consistent with the findings of others [33]. The relationships among production intensity, energy metabolism, maintenance of homeostasis and changes in FA proportions were confirmed by Młynek et al. [39]. The authors suggest focusing primarily on leptin and glucose, co-regulators of appetite and negative energy balance. Leptin correlated positively and significantly with non-esterified FAs (NEFA). It indicates that a higher level of the appetite reducing leptin accompanied a higher concentration of NEFAs released during the energy deficit.

Lactation parity did not show such a significant influence on the FA proportions; only C10:1, C12:0, C12:1, *iso*C14:0, C14:1n-5*c*, C16:1n-7*c*, C16:1, C17:0, C18:0, C18:1n-7*c*, C20:0, C20:1n-7*c*, C22:5n-3, PUFAn-3, LCFA, BCFA, and the sum of C18 isomers showed significant or significant close differences among lactations (Table 3, Table 4, Table 5, Table 6 and Table 7).

Genotype did not interact significantly with farm and lactation stage, and lactation parity did not interact with lactation stage, with a few exceptions (Table 3, Table 4, Table 5, Table 6 and Table 7). In contrast, the significant interactions between the farm and the lactation stage were numerous. All cows in the experiment were fed by maize silage, grass silage, hay and feed concentrates, but the feed management and quality could vary among farms. Many authors confirmed the effect of feeding strategy and cow energy status during lactation on milk fat composition [40,41] but, importantly, separation of feeding effect from lactation phase effect is complicated [42]. FA content in the milk is affected by cow´s diet, de novo synthesis in the mammary gland, rumen microbiome activity or releasing from the body fat [43,44]. The interaction between the farm and lactation stage may reflect the feeding strategy and body conditions of cows at individual farms. The differences between farms on various lactation stage levels were significant in many cases. The changes of MUFA and UFA content during high body fat mobilization was reported by Nogalski et al. [40]. The authors found the association between cow energy status and MUFA and UFA concentrations in milk during lactation. The results in our study suggest that significant effects of farm, lactation stage and also interaction between these effects may indicate the relationship between feed management and quality, body condition and different lactation stages during lactation.

## 4. Conclusions

Concisely, the results support the crucial effect of the farm, lactation stage and their interaction on the FA proportions of milk fat. The influence of lactation parity was markedly weaker and that of breed almost negligible. The effect of gene polymorphisms was different: for *AGPAT6* and *DGAT1* inconsiderable and *LEP* and *FASN* slightly higher, but nonsignificant FAs prevailed substantially too. For the individual FAs, the profile was most affected by *SCD1* polymorphism, and the effect of *AGPAT6* was negligible. The results hint at the intermediary heredity of the *SCD1* gene. Overall, of 60 FAs and groups, in 27 was found a significant effect of the gene polymorphism. With rare exceptions, the significant FAs did not overlap among genes. The interaction of farm x lactation stage showed a significant effect on the FA profile but not genotype x farm and lactation stage or lactation parity x stage. The data set was collected in the five chosen farms/milking parlors and was not specially modified to fit the blocked design. Therefore, some breeds and genes are not evenly distributed on individual farms. This fact could have slightly biased results in this analysis. Further, the differences in FA composition among genotypes were rather small, which could lead to overestimation of the effect and needs to be considered in the next research. Further studies of major genes effects on the FA profile are encouraged since elucidation of the genetic control is promising for future breeding.

## Figures and Tables

**Table 1:** Absolute frequencies of samples from cows with respective genotype ^1^.

*AGPAT6*			*DGAT1*		*LEP*			*FASN*		*SCD1*		
*CC*	*CT*	*TT*	*AA*	*AK*	*MM*	*MW*	*WW*	*AG*	*GG*	*CC*	*CT*	*TT*
256	560	14	816	44	542	130	26	206	652	226	546	84

^1^ *AGPAT6* = 1-acylglycerol-3-phosphate O-acyltransferase 6; *DGAT1* = acyl-CoA diacylglycerol transferase 1; *LEP* = leptin; *FASN* = fatty acid synthase; *SCD1* = stearoyl CoA desaturase 1.

**Table 2:** Significance of differences among genotypes on the fatty acid (FA) proportions (*p*-values) ^1^.

FAs and FA Groups ^2^		*AGPAT6*	*DGAT1*	*LEP*	*FASN*	*SCD1*
C10:0	4					0.010 *TT* ^A^ > *CT* ^a^ > *CC* ^Bb^
C10:1 *	5					<0.0001 *CC* ^A^ > *CT* ^B^ > *TT* ^C^
C12:0	7					0.028 *TT* ^a^ > *CT* ^a^ > *CC* ^b^
C12:1 *	8					<0.0001 *CC* ^Aa^ > *CT* ^Ab^ > *TT* ^B^
*iso*C14:0	10				0.045 * AG ^a^ > *GG* ^b^	
C14:0	11				0.050 *AG* > *GG*	0.007 *TT* ^Ab^ > *CT* ^a^ > *CC* ^Bb^
C14:1n-5*c*	12					<0.0001 *CC* ^A^ > *CT* ^B^ > *TT* ^C^
C15:0	14			0.062 *WW* > *MM* > *MW*		
*iso*C16:0	16				0.074 *AG* > *GG*	
C16:1n-7*c*	20					0.002 *TT* ^a^ > *CT* ^A^ > *CC* ^Bb^
*iso*C17:0	21					0.013 *CC* ^A^ > *CT* ^A^ > *TT* ^B^
C16:1 *	22				0.042 *AG* ^a^ > *GG* ^b^	0.026 *CC* ^A^ > *CT* ^a^ > *TT* ^Bb^
*anteiso*C17:0	23			0.011 *WW* ^Aa^ > *MW* ^b^ > *MM* ^B^		
C18:1n-7*t*	25	0.088 *TT* > *CT* > *CC*			0.075 *AG* > *GG*	
C18:1 *	32				0.082 *AG* > *GG*	0.018 *CC* ^a^ > *TT* ^b^ > *CT* ^b^
C18:2 *	33					0.0006 *TT* ^A^ > *CT* ^A^ > *CC* ^B^
C19:1 *	35			0.063 *WW* > *MW*, *MM*		
C18:3 *	36			0.0002 *WW* ^A^ > *MW* ^B^, *MM* ^B^		
C20:1n-9*c*	40				0.046 *AG* ^a^ > *GG* ^b^	
C20:4n-6 **	44	0.095 *TT* > *CT* > *CC*		0.006 *WW* ^Aa^ > *MW* ^b^ > *MM* ^B^		
C22:0	45		0.030 *AA* ^a^ > *AK* ^b^			
C20:4n-3 **	46		0.003 *AK* ^A^ > *AA* ^B^		0.066 *AG* > *GG*	
C20:5n-3 **	47			0.042 *WW* ^a^ > *MM* ^b^, *MW* ^b^		
C24:0	48	0.096 *CT* > *TT* > *CC*				
C22:5n-3 **	49			0.005 *WW* ^A^ > *MM* ^B^, *MW* ^B^	0.021 *AG* ^a^ > *GG* ^b^	
PUFAn-3	54			0.059 *WW* > *MM* > *MW*		
BCFA	59			0.029 *WW* ^a^ > *MW* > *MM* ^b^	0.048 *AG* ^a^ > *GG* ^b^	

^a,b^ different letters between genotypes in the same gene and FA represent significant differences at *p* < 0.05; ^A,B,C^ different letters between genotypes in the same gene and FA represent significant differences at *p* < 0.01; ^1^ *p*-value = *p*-value of the polymorphism effect; only *p*-values of FAs with significant effects of genotype are given, and FAs with *p-*value near to the significance threshold; in other FAs the effect of genotype was not significant, i.e., in C4:0 (1), C6:0 (2), C8:0 (3), C11:0 (6), C13:0 (9), *anteiso*C15:0 (13), C15:1 * (15), C16:0 (17), C16:1 * (18), C16:1 * (19), C17:0 (24), C17:1n-7*c* (25), C18:0 (26), C18:1*t* * (27), C18:1n-9*c*, C18:1n-7*c* (30), C18:1 * (31), C18:2n-6 ** (34), C18:3n-3 ** (37), C20:0 (38), C18:2*c*9,*t*11 (39), C20:1n-7*c* (41), C21:0 (42), C20:3n-6 ** (43), saturated FAs (50), monounsaturated FAs (51), *trans* isomers of unsaturated FAs (52), polyunsaturated FAs (53), short-chain FAs (55), medium-chain FAs (56), long-chain FAs (57), unsaturated FA (58), the sum of FA with C18 (60); the number in brackets indicates the order, see also Table S2. ^2^ FA proportions were stated as g/100 g of FAs; * = unidentified position isomer; ** = all-*cis* isomer; *c* = *cis* isomer; *t* = *trans* isomer; PUFAn-3 = the sum of polyunsaturated FAs n-3; BCFA = branched-chain FAs.

**Table 3:** Effects on the fatty acid (FA) proportions (*p-*values)—*AGPAT6* gene.

FAs and FA Groups ^1^		Effects					Interaction			
		Farm	Gene	Lactation Parity	Lactation Stage	Breed of the Cow	Genotype * Farm	Genotype * Lactation Stage	Farm * Lactation Stage	Lactation Parity * Lactation Stage
C4:0	1	***	ns	ns	***	ns	ns	ns	**	ns
C6:0	2	***	ns	ns	**	ns	N/A	N/A	*	ns
C8:0	3	***	ns	ns	ns	ns	ns	ns	**	ns
C10:0	4	***	ns	ns	ns	ns	N/A	N/A	**	ns
C10:1 *	5	***	ns	ns	***	ns	ns	ns	ns	ns
C11:0	6	***	ns	ns	**	ns	ns	ns	*	ns
C12:0	7	**	ns	ns	**	ns	ns	ns	**	ns
C12:1 *	8	*	ns	*	***	ns	N/A	ns	ns	ns
C13:0	9	***	ns	ns	***	ns	ns	ns	ns	ns
*iso*C14:0	10	*	ns	*	**	*	ns	ns	ns	ns
C14:0	11	***	ns	ns	***	ns	N/A	ns	ns	ns
C14:1n-5*c*	12	***	ns	*	***	**	N/A	ns	ns	ns
*anteiso*C15:0	13	***	ns	ns	**	ns	ns	ns	ns	ns
C15:0	14	***	ns	ns	***	ns	ns	ns	ns	ns
C15:1 *	15	***	ns	ns	ns	ns	N/A	ns	***	*
*iso*C16:0	16	***	ns	ns	*	*	ns	ns	ns	ns
C16:0	17	***	ns	ns	***	ns	ns	ns	**	ns
C16:1 *	18	***	ns	ns	***	ns	ns	ns	***	ns
C16:1 *	19	***	ns	ns	***	ns	N/A	ns	ns	ns
C16:1n-7*c*	20	ns	ns	*	*	*	N/A	ns	**	ns
*iso*C17:0	21	***	ns	ns	**	ns	ns	ns	ns	ns
C16:1 *	22	**	ns	***	***	ns	ns	ns	ns	ns
*anteiso*C17:0	23	***	ns	ns	ns	ns	ns	ns	ns	ns
C17:0	24	***	ns	ns	***	ns	ns	ns	ns	ns
C17:1n-7*c*	25	***	ns	ns	***	ns	N/A	ns	***	ns
C18:0	26	***	ns	*	***	ns	N/A	ns	**	ns
C18:1t *	27	***	ns	ns	***	ns	ns	ns	ns	ns
C18:1n-7*t*	28	***	ns	ns	***	ns	ns	ns	**	ns
C18:1n-9*c*	29	***	ns	ns	***	ns	ns	ns	**	ns
C18:1n-7*c*	30	***	ns	ns	***	ns	N/A	ns	ns	ns
C18:1 *	31	***	ns	ns	***	ns	N/A	ns	**	ns
C18:1 *	32	***	ns	ns	***	ns	N/A	ns	ns	ns
C18:2 *	33	***	ns	ns	***	ns	N/A	ns	ns	ns
C18:2n-6 **	34	***	ns	ns	ns	ns	ns	ns	**	ns
C19:1 *	35	***	ns	ns	**	ns	N/A	ns	***	ns
C18:3 *	36	***	ns	ns	ns	ns	N/A	N/A	ns	**
C18:3n-3 **	37	***	ns	ns	*	ns	N/A	ns	ns	ns
C20:0	38	***	ns	**	***	*	N/A	ns	ns	ns
C18:2*c*9,*t*11	39	***	ns	ns	***	ns	ns	ns	*	ns
C20:1n-9*c*	40	***	ns	ns	***	ns	ns	ns	*	ns
C20:1n-7*c*	41	***	ns	ns	***	ns	ns	ns	**	ns
C21:0	42	***	ns	ns	ns	ns	ns	ns	ns	ns
C20:3n-6 **	43	***	ns	ns	**	ns	N/A	ns	ns	ns
C20:4n-6 **	44	***	ns	ns	ns	ns	N/A	ns	***	ns
C22:0	45	***	ns	ns	**	**	ns	ns	*	ns
C20:4n-3 **	46	***	ns	ns	**	*	*	ns	ns	ns
C20:5n-3 **	47	***	ns	ns	***	ns	ns	ns	ns	ns
C24:0	48	***	ns	ns	*	ns	ns	ns	ns	***
C22:5n-3 **	49	***	ns	***	***	ns	ns	ns	ns	ns
SFA	50	***	ns	ns	*	ns	ns	ns	*	ns
MUFA	51	***	ns	ns	**	ns	ns	ns	**	ns
TFA	52	***	ns	ns	***	ns	N/A	ns	*	ns
PUFA	53	***	ns	ns	ns	ns	N/A	ns	ns	ns
PUFAn-3	54	***	ns	*	***	ns	ns	ns	ns	ns
SCFA	55	***	ns	ns	ns	ns	ns	ns	**	ns
MCFA	56	***	ns	ns	***	ns	N/A	ns	**	ns
LCFA	57	***	ns	ns	***	ns	ns	ns	*	ns
UFA	58	***	ns	ns	*	ns	N/A	N/A	*	ns
BCFA	59	***	ns	*	**	ns	ns	ns	ns	ns
C18	60	***	ns	*	***	ns	ns	N/A	*	*

* = significant at *p* < 0.05; ** = significant at *p* < 0.01; *** = significant at *p* < 0.001; ns = not significant. N/A Not Applicable due to the low number of samples. ^1^ * = unidentified position isomer; ** = all-*cis* isomer; *c = cis* isomer; *t = trans* isomer; SFA = saturated FA; MUFA = monounsaturated FA; TFA = *trans* isomers of unsaturated FA; PUFA = polyunsaturated FA; PUFAn-3 = the sum of polyunsaturated FA n-3; SCFA = short-chain FA; MCFA = medium-chain FA; LCFA = long-chain FA; UFA = unsaturated FA; BCFA = branched-chain FA; C18 = the sum of FA with C18.

**Table 4:** Effects on the fatty acid (FA) proportions (*p-*values)—*DGAT1* gene.

FAs and FA Groups ^1^		Effects					Interaction			
		Farm	Gene	Lactation Parity	Lactation Stage	Breed of the Cow	Genotype * Farm	Genotype * Lactation Stage	Farm * Lactation Stage	Lactation Parity * Lactation Stage
C4:0	1	***	ns	***	ns	ns	*	**	**	ns
C6:0	2	***	ns	*	ns	ns	**	*	*	ns
C8:0	3	***	ns	ns	ns	ns	*	**	**	ns
C10:0	4	**	ns	ns	ns	ns	*	**	**	ns
C10:1 *	5	***	*	***	ns	ns	*	ns	ns	ns
C11:0	6	***	ns	**	ns	ns	ns	*	*	ns
C12:0	7	**	*	**	ns	ns	*	**	**	ns
C12:1 *	8	*	*	***	ns	ns	ns	ns	ns	ns
C13:0	9	***	ns	***	ns	ns	ns	ns	ns	ns
*iso*C14:0	10	*	ns	*	***	*	ns	ns	ns	ns
C14:0	11	***	ns	ns	***	ns	ns	ns	ns	ns
C14:1n-5*c*	12	***	ns	*	***	**	ns	ns	ns	ns
*anteiso*C15:0	13	***	ns	ns	**	ns	ns	ns	ns	ns
C15:0	14	***	ns	ns	***	ns	ns	ns	ns	ns
C15:1 *	15	***	ns	ns	ns	ns	ns	ns	***	*
*iso*C16:0	16	***	ns	ns	*	*	ns	ns	ns	ns
C16:0	17	***	ns	ns	***	*	ns	ns	**	ns
C16:1 *	18	***	ns	ns	***	ns	ns	ns	***	*
C16:1*	19	***	ns	ns	***	ns	ns	ns	ns	ns
C16:1n-7*c*	20	ns	ns	*	*	*	ns	ns	*	ns
*iso*C17:0	21	***	ns	ns	**	ns	ns	ns	ns	ns
C16:1 *	22	**	ns	***	***	ns	ns	ns	ns	ns
*anteiso*C17:0	23	***	ns	ns	ns	ns	ns	ns	*	ns
C17:0	24	***	ns	ns	***	ns	ns	ns	ns	ns
C17:1n-7*c*	25	***	ns	ns	***	ns	ns	**	***	ns
C18:0	26	***	ns	*	***	ns	ns	ns	*	ns
C18:1t *	27	***	ns	ns	***	ns	ns	ns	ns	ns
C18:1n-7*t*	28	***	ns	ns	***	ns	ns	ns	***	ns
C18:1n-9*c*	29	***	ns	ns	***	ns	ns	ns	**	ns
C18:1n-7*c*	30	***	ns	ns	***	ns	ns	ns	ns	ns
C18:1 *	31	***	ns	ns	***	ns	ns	ns	***	ns
C18:1 *	32	***	ns	ns	**	ns	ns	ns	ns	ns
C18:2 *	33	***	ns	ns	***	ns	ns	ns	ns	ns
C18:2n-6 **	34	***	ns	ns	ns	ns	ns	ns	**	ns
C19:1 *	35	***	ns	ns	**	ns	ns	*	***	ns
C18:3 *	36	***	ns	ns	ns	ns	***	ns	ns	**
C18:3n-3 **	37	***	ns	ns	*	ns	ns	ns	ns	ns
C20:0	38	***	ns	**	***	ns	ns	ns	ns	ns
C18:2*c*9,*t*11	39	***	ns	ns	***	ns	ns	ns	*	ns
C20:1n-9*c*	40	***	ns	ns	***	ns	ns	ns	**	ns
C20:1n-7*c*	41	***	ns	ns	***	ns	*	ns	**	ns
C21:0	42	***	ns	ns	ns	ns	ns	ns	ns	ns
C20:3n-6 **	43	**	ns	ns	**	ns	**	ns	ns	ns
C20:4n-6 **	44	**	ns	ns	ns	ns	ns	ns	***	ns
C22:0	45	***	*	ns	**	*	ns	ns	*	ns
C20:4n-3 **	46	***	**	ns	**	*	ns	**	ns	ns
C20:5n-3 **	47	***	ns	ns	***	ns	ns	ns	ns	ns
C24:0	48	***	ns	ns	*	ns	ns	ns	ns	**
C22:5n-3 **	49	***	ns	***	***	ns	ns	ns	*	ns
SFA	50	***	ns	ns	**	ns	ns	ns	*	ns
MUFA	51	***	ns	ns	**	ns	ns	ns	**	ns
TFA	52	***	ns	ns	***	ns	ns	ns	*	ns
PUFA	53	***	ns	ns	ns	ns	ns	ns	ns	ns
PUFAn-3	54	***	ns	**	***	ns	ns	ns	ns	*
SCFA	55	***	ns	ns	ns	ns	ns	**	**	ns
MCFA	56	***	ns	ns	***	ns	ns	ns	**	ns
LCFA	57	***	ns	*	***	ns	ns	ns	*	ns
UFA	58	***	ns	ns	**	ns	ns	ns	*	ns
BCFA	59	***	ns	*	***	ns	ns	ns	ns	*
C18	60	***	ns	*	***	ns	ns	ns	**	ns

* = significant at *p* < 0.05; ** = significant at *p* < 0.01; *** = significant at *p* < 0.001; ns = not significant. ^1^ * = unidentified position isomer; ** = all-*cis* isomer; *c = cis* isomer; *t = trans* isomer; SFA = saturated FA; MUFA = monounsaturated FA; TFA = *trans* isomers of unsaturated FA; PUFA = polyunsaturated FA; PUFAn-3 = the sum of polyunsaturated FA n-3; SCFA = short-chain FA; MCFA = medium-chain FA; LCFA = long-chain FA; UFA = unsaturated FA; BCFA = branched-chain FA; C18 = the sum of FA with C18.

**Table 5:** Effects on the fatty acid (FA) proportions (*p-*values)—*FASN* gene.

FAs and FA Groups ^1^		Effects					Interaction			
		Farm	Gene	Lactation Parity	Lactation Stage	Breed of the Cow	Genotype * Farm	Genotype * Lactation Stage	Farm * Lactation Stage	Lactation Parity * Lactation Stage
C4:0	1	***	ns	ns	***	ns	ns	ns	**	ns
C6:0	2	***	ns	ns	*	ns	ns	ns	*	ns
C8:0	3	***	ns	ns	ns	ns	ns	ns	**	ns
C10:0	4	**	ns	ns	ns	ns	ns	ns	**	ns
C10:1 *	5	***	ns	*	***	ns	ns	ns	**	ns
C11:0	6	***	ns	ns	**	ns	ns	ns	*	ns
C12:0	7	***	ns	*	**	ns	ns	ns	**	ns
C12:1 *	8	ns	ns	*	***	ns	ns	ns	*	ns
C13:0	9	***	ns	ns	***	ns	ns	ns	ns	ns
*iso*C14:0	10	ns	*	ns	**	*	ns	ns	ns	ns
C14:0	11	***	ns	ns	***	ns	ns	ns	ns	ns
C14:1n-5*c*	12	***	ns	*	***	*	ns	ns	*	ns
*anteiso*C15:0	13	***	ns	ns	**	ns	ns	ns	ns	ns
C15:0	14	***	ns	ns	***	ns	ns	ns	ns	ns
C15:1 *	15	***	ns	ns	ns	ns	ns	ns	***	*
*iso*C16:0	16	***	ns	ns	*	*	ns	ns	ns	ns
C16:0	17	***	ns	ns	***	*	ns	ns	**	ns
C16:1 *	18	***	ns	ns	***	ns	ns	ns	***	*
C16:1 *	19	***	ns	ns	***	ns	ns	ns	ns	ns
C16:1n-7*c*	20	ns	ns	*	ns	*	ns	ns	**	ns
*iso*C17:0	21	***	ns	ns	**	ns	ns	ns	ns	ns
C16:1 *	22	***	*	***	***	ns	ns	ns	*	ns
*anteiso*C17:0	23	***	ns	ns	ns	ns	ns	ns	ns	ns
C17:0	24	***	ns	*	***	ns	ns	ns	ns	ns
C17:1n-7*c*	25	***	ns	ns	***	*	*	ns	***	ns
C18:0	26	***	ns	**	***	ns	ns	ns	***	ns
C18:1t *	27	***	ns	ns	***	ns	ns	ns	*	ns
C18:1n-7*t*	28	***	ns	ns	***	ns	ns	ns	***	ns
C18:1n-9*c*	29	***	ns	ns	***	ns	ns	ns	**	ns
C18:1n-7*c*	30	***	ns	ns	***	ns	ns	ns	**	ns
C18:1 *	31	***	ns	ns	***	ns	ns	ns	***	ns
C18:1 *	32	***	ns	ns	**	ns	ns	ns	*	ns
C18:2 *	33	***	ns	ns	***	ns	ns	ns	ns	ns
C18:2n-6 **	34	***	ns	ns	ns	ns	*	ns	**	ns
C19:1 *	35	***	ns	ns	**	ns	**	ns	***	ns
C18:3 *	36	***	ns	ns	ns	ns	ns	ns	ns	**
C18:3n-3 **	37	***	ns	ns	*	ns	ns	ns	ns	ns
C20:0	38	***	ns	**	***	*	*	ns	*	ns
C18:2*c*9,*t*11	39	***	ns	ns	***	ns	ns	ns	ns	ns
C20:1n-9*c*	40	***	*	ns	***	ns	ns	ns	*	ns
C20:1n-7*c*	41	***	ns	*	***	ns	ns	ns	**	ns
C21:0	42	***	ns	ns	ns	ns	ns	ns	ns	ns
C20:3n-6 **	43	**	ns	ns	**	ns	ns	ns	ns	*
C20:4n-6 **	44	**	ns	ns	ns	ns	ns	ns	***	ns
C22:0	45	***	ns	ns	**	*	ns	ns	*	ns
C20:4n-3 **	46	***	ns	ns	**	**	ns	ns	ns	*
C20:5n-3 **	47	***	ns	ns	***	ns	ns	ns	ns	ns
C24:0	48	***	ns	ns	*	ns	ns	ns	ns	**
C22:5n-3 **	49	***	*	***	***	ns	ns	ns	ns	ns
SFA	50	***	ns	ns	**	ns	ns	ns	*	ns
MUFA	51	***	ns	ns	**	ns	ns	ns	**	ns
TFA	52	***	ns	ns	***	ns	ns	ns	*	ns
PUFA	53	***	ns	ns	ns	ns	ns	ns	ns	ns
PUFAn-3	54	***	ns	**	***	ns	ns	ns	ns	**
SCFA	55	***	ns	ns	ns	ns	ns	ns	**	ns
MCFA	56	***	ns	*	***	ns	ns	ns	***	ns
LCFA	57	***	ns	*	***	ns	ns	ns	**	ns
UFA	58	***	ns	ns	**	ns	ns	ns	*	ns
BCFA	59	***	*	*	**	ns	ns	ns	ns	*
C18	60	***	ns	*	***	ns	ns	ns	**	ns

* = significant at *p* < 0.05; ** = significant at *p* < 0.01; *** = significant at *p* < 0.001; ns = not significant. ^1^ * = unidentified position isomer; ** = all-*cis* isomer; *c = cis* isomer; *t = trans* isomer; SFA = saturated FA; MUFA = monounsaturated FA; TFA = *trans* isomers of unsaturated FA; PUFA = polyunsaturated FA; PUFAn-3 = the sum of polyunsaturated FA n-3; SCFA = short-chain FA; MCFA = medium-chain FA; LCFA = long-chain FA; UFA = unsaturated FA; BCFA = branched-chain FA; C18 = the sum of FA with C18.

**Table 6:** Effects on the fatty acid (FA) proportions (*p-*values)—*LEP* gene.

FAs and FA Groups ^1^		Effects					Interaction			
		Farm	Gene	Lactation Parity	Lactation Stage	Breed of the Cow	Genotype * Farm	Genotype * Lactation Stage	Farm * Lactation Stage	Lactation Parity * Lactation Stage
C4:0	1	***	ns	ns	***	ns	ns	ns	***	ns
C6:0	2	***	ns	ns	*	ns	ns	ns	*	ns
C8:0	3	***	ns	ns	ns	ns	ns	*	**	ns
C10:0	4	***	ns	ns	ns	ns	ns	ns	***	ns
C10:1 *	5	***	ns	**	***	ns	ns	ns	***	ns
C11:0	6	***	ns	ns	*	ns	ns	*	**	ns
C12:0	7	***	ns	ns	**	ns	ns	ns	***	ns
C12:1 *	8	**	ns	*	***	ns	ns	*	**	ns
C13:0	9	***	ns	ns	***	ns	ns	**	*	ns
*iso*C14:0	10	ns	ns	ns	***	*	ns	ns	ns	ns
C14:0	11	***	ns	ns	***	ns	ns	ns	*	ns
C14:1n-5*c*	12	***	ns	*	***	**	ns	ns	*	ns
*anteiso*C15:0	13	***	ns	ns	**	ns	ns	ns	ns	ns
C15:0	14	***	ns	ns	***	ns	ns	*	ns	ns
C15:1 *	15	***	ns	ns	ns	ns	ns	ns	***	*
*iso*C16:0	16	**	ns	ns	*	*	ns	ns	ns	ns
C16:0	17	***	ns	ns	***	ns	ns	ns	**	ns
C16:1*	18	***	ns	ns	***	ns	ns	ns	***	ns
C16:1 *	19	***	ns	ns	***	ns	ns	ns	ns	ns
C16:1n-7*c*	20	ns	ns	ns	ns	*	ns	ns	*	ns
*iso*C17:0	21	***	ns	ns	**	ns	ns	ns	ns	ns
C16:1*	22	***	ns	***	***	ns	ns	ns	**	ns
*anteiso*C17:0	23	***	*	ns	ns	ns	ns	ns	ns	ns
C17:0	24	***	ns	ns	***	ns	ns	ns	ns	ns
C17:1n-7*c*	25	***	ns	ns	***	ns	ns	ns	***	ns
C18:0	26	***	ns	**	***	ns	ns	ns	***	ns
C18:1t *	27	***	ns	ns	***	ns	ns	ns	ns	ns
C18:1n-7*t*	28	***	ns	ns	***	ns	ns	ns	**	ns
C18:1n-9*c*	29	***	ns	ns	***	ns	ns	ns	***	ns
C18:1n-7*c*	30	***	ns	*	***	ns	ns	ns	ns	ns
C18:1 *	31	***	ns	ns	***	ns	ns	ns	*	ns
C18:1 *	32	***	ns	ns	***	ns	ns	ns	ns	ns
C18:2 *	33	***	ns	ns	***	ns	ns	ns	ns	ns
C18:2n-6 **	34	***	ns	ns	ns	ns	ns	ns	**	*
C19:1 *	35	***	ns	ns	*	ns	ns	ns	***	ns
C18:3 *	36	***	***	ns	ns	ns	***	ns	ns	**
C18:3n-3 **	37	***	ns	ns	*	ns	ns	ns	ns	ns
C20:0	38	***	ns	**	**	*	ns	ns	*	ns
C18:2*c*9,*t*11	39	***	ns	ns	***	ns	ns	ns	*	ns
C20:1n-9*c*	40	***	ns	ns	***	ns	ns	ns	*	ns
C20:1n-7*c*	41	***	ns	*	***	ns	*	ns	*	ns
C21:0	42	***	ns	ns	ns	ns	ns	ns	ns	ns
C20:3n-6 **	43	*	ns	ns	**	ns	**	ns	ns	ns
C20:4n-6 **	44	*	**	ns	ns	ns	ns	ns	***	ns
C22:0	45	***	ns	ns	*	*	ns	ns	*	ns
C20:4n-3 **	46	***	ns	ns	*	*	ns	ns	ns	*
C20:5n-3 **	47	***	*	ns	***	ns	ns	ns	ns	ns
C24:0	48	***	ns	ns	*	ns	ns	ns	ns	ns
C22:5n-3 **	49	***	**	***	***	ns	ns	ns	*	*
SFA	50	***	ns	ns	*	ns	ns	ns	*	*
MUFA	51	***	ns	ns	*	ns	ns	ns	***	***
TFA	52	***	ns	ns	***	ns	ns	ns	*	*
PUFA	53	***	ns	ns	ns	ns	ns	ns	ns	ns
PUFAn-3	54	***	ns	*	***	ns	ns	ns	ns	*
SCFA	55	***	ns	ns	ns	ns	ns	*	**	ns
MCFA	56	***	ns	ns	***	ns	ns	ns	***	ns
LCFA	57	***	ns	*	***	ns	ns	ns	***	ns
UFA	58	***	ns	ns	*	ns	ns	ns	**	ns
BCFA	59	***	*	*	***	ns	ns	ns	*	*
C18	60	***	ns	*	***	ns	ns	ns	***	ns

* = significant at *p* < 0.05; ** = significant at *p* < 0.01; *** = significant at *p* < 0.001; ns = not significant. ^1^ * = unidentified position isomer; ** = all-*cis* isomer; *c = cis* isomer; *t = trans* isomer; SFA = saturated FA; MUFA = monounsaturated FA; TFA = *trans* isomers of unsaturated FA; PUFA = polyunsaturated FA; PUFAn-3 = the sum of polyunsaturated FA n-3; SCFA = short-chain FA; MCFA = medium-chain FA; LCFA = long-chain FA; UFA = unsaturated FA; BCFA = branched-chain FA; C18 = the sum of FA with C18.

**Table 7:** Effects on the fatty acid (FA) proportions (*p-*values)—*SCD1* gene.

FAs and FA Groups ^1^		Effects					Interaction			
		Farm	Gene	Lactation Parity	Lactation Stage	Breed of the Cow	Genotype * Farm	Genotype * Lactation Stage	Farm * Lactation Stage	Lactation Parity * Lactation Stage
C4:0	1	***	ns	ns	***	ns	ns	ns	**	ns
C6:0	2	***	ns	ns	*	ns	ns	ns	*	ns
C8:0	3	***	ns	ns	ns	ns	ns	ns	**	ns
C10:0	4	**	*	ns	ns	ns	ns	ns	**	ns
C10:1 *	5	***	***	*	***	ns	ns	ns	**	ns
C11:0	6	***	ns	ns	**	ns	ns	ns	*	ns
C12:0	7	**	*	*	**	ns	ns	ns	**	ns
C12:1 *	8	*	***	*	***	ns	ns	ns	*	ns
C13:0	9	***	ns	ns	***	ns	ns	ns	ns	ns
*iso*C14:0	10	ns	ns	*	***	*	ns	ns	ns	ns
C14:0	11	ns	**	ns	***	ns	ns	ns	ns	ns
C14:1n-5*c*	12	ns	***	*	***	*	ns	ns	ns	ns
*anteiso*C15:0	13	ns	ns	ns	**	ns	ns	ns	ns	ns
C15:0	14	ns	ns	ns	***	ns	ns	ns	ns	ns
C15:1 *	15	ns	ns	ns	ns	ns	ns	ns	***	*
*iso*C16:0	16	ns	ns	ns	*	*	ns	ns	ns	ns
C16:0	17	ns	ns	ns	***	*	ns	ns	**	ns
C16:1 *	18	ns	ns	ns	***	ns	ns	ns	***	*
C16:1 *	19	ns	ns	ns	***	ns	ns	*	ns	ns
C16:1n-7*c*	20	ns	**	*	ns	*	ns	ns	***	ns
*iso*C17:0	21	***	*	ns	**	ns	ns	ns	ns	ns
C16:1 *	22	**	*	***	***	ns	ns	ns	ns	ns
*anteiso*C17:0	23	***	ns	ns	ns	ns	ns	ns	ns	*
C17:0	24	***	ns	*	***	ns	ns	ns	ns	ns
C17:1n-7*c*	25	***	ns	ns	***	ns	ns	ns	***	ns
C18:0	26	***	ns	**	***	ns	ns	ns	***	ns
C18:1t *	27	***	ns	ns	***	ns	ns	ns	*	ns
C18:1n-7*t*	28	***	ns	ns	***	ns	ns	ns	***	ns
C18:1n-9*c*	29	***	ns	ns	***	ns	ns	ns	**	ns
C18:1n-7*c*	30	***	ns	*	***	ns	ns	ns	ns	ns
C18:1 *	31	***	ns	ns	***	ns	ns	ns	***	ns
C18:1 *	32	***	*	ns	**	ns	ns	ns	*	ns
C18:2 *	33	***	***	ns	***	ns	ns	ns	ns	ns
C18:2n-6 **	34	***	ns	ns	ns	ns	ns	ns	**	ns
C19:1 *	35	***	ns	ns	*	ns	ns	ns	***	*
C18:3 *	36	***	ns	ns	ns	ns	ns	*	ns	**
C18:3n-3 **	37	***	ns	ns	*	ns	ns	ns	ns	ns
C20:0	38	***	ns	**	***	*	ns	ns	*	ns
C18:2*c*9,*t*11	39	***	ns	ns	***	ns	ns	ns	*	ns
C20:1n-9*c*	40	***	ns	ns	***	ns	ns	ns	*	ns
C20:1n-7*c*	41	***	ns	ns	***	ns	ns	ns	**	ns
C21:0	42	***	ns	ns	ns	ns	ns	ns	ns	ns
C20:3n-6 **	43	**	ns	ns	**	ns	**	ns	ns	ns
C20:4n-6 **	44	**	ns	ns	ns	ns	ns	ns	***	ns
C22:0	45	***	ns	ns	*	*	ns	ns	*	ns
C20:4n-3 **	46	***	ns	ns	**	*	ns	ns	ns	*
C20:5n-3 **	47	***	ns	ns	***	ns	*	ns	ns	ns
C24:0	48	***	ns	ns	*	ns	ns	ns	ns	**
C22:5n-3 **	49	***	ns	***	***	ns	ns	ns	ns	ns
SFA	50	***	ns	ns	**	ns	ns	ns	*	ns
MUFA	51	***	ns	ns	**	ns	ns	ns	**	ns
TFA	52	***	ns	ns	***	ns	ns	ns	**	ns
PUFA	53	***	ns	ns	ns	ns	ns	ns	ns	ns
PUFAn-3	54	***	ns	**	***	ns	ns	ns	ns	**
SCFA	55	***	ns	ns	ns	ns	ns	ns	**	ns
MCFA	56	***	ns	ns	***	ns	ns	ns	**	ns
LCFA	57	***	ns	*	***	ns	ns	ns	**	ns
UFA	58	***	ns	ns	**	ns	ns	ns	*	ns
BCFA	59	***	ns	*	**	ns	ns	ns	ns	*
C18	60	***	ns	*	***	ns	ns	ns	**	ns

* = significant at *p* < 0.05; ** = significant at *p* < 0.01; *** = significant at *p* < 0.001; ns = not significant. ^1^ * = unidentified position isomer; ** = all-*cis* isomer; *c = cis* isomer; *t = trans* isomer; SFA = saturated FA; MUFA = monounsaturated FA; TFA = *trans* isomers of unsaturated FA; PUFA = polyunsaturated FA; PUFAn-3 = the sum of polyunsaturated FA n-3; SCFA = short-chain FA; MCFA = medium-chain FA; LCFA = long-chain FA; UFA = unsaturated FA; BCFA = branched-chain FA; C18 = the sum of FA with C18.

## Data Availability

The datasets generated for this study are available on reasonable request to the corresponding author.

## Supplementary Materials

The following are available online at https://www.mdpi.com/article/10.3390/ani11113284/s1, Table S1: Sequences of primers used in the polymerase chain reactions and restriction endonucleases used for genotyping, Table S2: Proportion of fatty acids (FAs) in different genotypes (LSM ± SE, *p*-value ^1^).

## References

[1] Hossein-Zadeh N.G. (2021). A meta-analysis of heritability estimates for milk fatty acids and their genetic relationship with milk production traits in dairy cows using a random-effects model. Livest. Sci..

[2] Lopez-Villalobos N., Spelman R.J., Melis J., Davis S.R., Berry S.D., Lehnert K., Sneddon N.W., Holroyd S.E., MacGibbon A.K., Snell R.G. (2020). Genetic correlations of milk fatty acid contents predicted from milk mid-infrared spectra in New Zealand dairy cattle. J. Dairy Sci..

[3] Park C.H., Ranaraja U., Dang C.G., Kim J.J., Do C.H. (2020). Genetic parameters for milk fatty acid composition of Holstein in Korea. Asian Australas. J. Anim. Sci..

[4] Bobbo T., Penasa M., Cassandro M. (2020). Genetic parameters of bovine milk fatty acid profile, yield, composition, total and differential somatic cell count. Animals.

[5] Manuelian C.L., Penasa M., Visentin G., Benedet A., Cassandro M., De Marchi M. (2019). Multi-breed herd approach to detect breed differences in composition and fatty acid profile of cow milk. Czech J. Anim. Sci..

[6] Safina N.Y., Shakirov S.K., Ravilov R.K., Sharafutdinov G.S. (2020). Associations of the *SCD1* gene SNP with fatty acids composition of Holstein cows. BIO Web Conf..

[7] Li C., Sun D.X., Zhang S.L., Yang S.H., Alim M.A., Zhang Q., Li Y.H., Liu L. (2016). Genetic effects of *FASN*, *PPARGC1A*, *ABCG2* and *IGF1* revealing the association with milk fatty acids in a Chinese Holstein cattle population based on a post genome-wide association study. BMC Genet..

[8] Klímová A., Kašná E., Machová K., Brzáková M., Přibyl J., Vostrý L. (2020). The use of genomic data and imputation methods in dairy cattle breeding. Czech J. Anim. Sci..

[9] Palombo V., Pegolo S., Conte G., Cesarani A., Macciotta N.P.P., Stefanon B., Marsan P.A., Mele M., Cecchinato A., D’Andrea M. (2021). Genomic prediction for latent variables related to milk fatty acid composition in Holstein, Simmental and Brown Swiss dairy cattle breeds. J. Anim. Breed. Genet..

[10] Maurić M., Mašek T., Ljoljić D.B., Grbavac J., Starčević K. (2019). Effects of different variants of the *FASN* gene on production performance and milk fatty acid composition in Holstein × Simmental dairy cows. Vet. Med.-Czech.

[11] Bordonaro S., Tumino S., Marletta D., De Angelis A., Di Paola F., Avondo M., Valenti B. (2020). Effect of *GH* p.L127V polymorphism and feeding systems on milk production traits and fatty acid composition in Modicana cows. Animals.

[12] Haruna I.L., Li Y.H., Ekegbu U.J., Amirpour-Najafabadi H., Zhou H.T., Hickford J.G.H. (2020). Associations between the bovine myostatin gene and milk fatty acid composition in New Zealand Holstein-Friesian x Jersey-cross cows. Animals.

[13] Proskura W.S., Liput M., Zaborski D., Sobek Z., Yu Y.H., Cheng Y.H., Dybus A. (2019). The effect of polymorphism in the *FADS2* gene on the fatty acid composition of bovine milk. Arch. Anim. Breed..

[14] Shi L., Liu L., Lv X., Ma Z., Li C., Li Y., Zhao F., Sun D., Han B. (2020). Identification of genetic effects and potential causal polymorphisms of *CPM* gene impacting milk fatty acid traits in Chinese Holstein. Anim. Genet..

[15] Azis R., Jakaria, Anggraeni A., Gunawan A. (2020). Acetyl-CoA carboxylase alpha gene polymorphism and its association with milk fatty acid of Holstein Friesian using real-time PCR method. Trop. Anim. Sci. J..

[16] Polasik D., Golinczak J., Proskura W., Terman A., Dybus A. (2021). Association between *THRSP* gene polymorphism and fatty acid composition in milk of dairy cows. Animals.

[17] Shi L.J., Wu X., Yang Y.Z., Ma Z., Lv X.Q., Liu L., Li Y.H., Zhao F., Han B., Sun D.X. (2021). A post-GWAS confirming the genetic effects and functional polymorphisms of *AGPAT3* gene on milk fatty acids in dairy cattle. J. Anim. Sci. Biotechnol..

[18] Čítek J., Hanusová L., Brzáková M., Večerek L., Panicke L., Lískovcová L. (2018). Associations between gene polymorphisms, breeding values, and glucose tolerance test parameters in German Holstein sires. Czech J. Anim. Sci..

[19] Kuehn C., Edel C., Weikard R., Thaller G. (2007). Dominance and parent-of-origin effects of coding and non-coding alleles at the acylCoA-diacylglycerol-acyltransferase (*DGATI*) gene on milk production traits in German Holstein cows. BMC Genet..

[20] Carvajal A.M., Huircan P., Dezamour J.M., Subiabre I., Kerr B., Morales R., Ungerfeld E.M. (2016). Milk fatty acid profile is modulated by *DGAT1* and *SCD1* genotypes in dairy cattle on pasture and strategic supplementation. Genet. Mol. Res..

[21] Inostroza K.B., Scheuermann E.S., Sepulveda N.A. (2013). Stearoyl CoA desaturase and fatty acid synthase gene polymorphisms and milk fatty acid composition in Chilean Black Friesian cows. Rev. Colomb. Cienc. Pec..

[22] Samková E., Špička J., Hanuš O., Roubal P., Pecová L., Hasoňová L., Smetana P., Klimešová M., Čítek J. (2020). Comparison of fatty acid proportions determined by mid-infrared spectroscopy and gas chromatography in bulk and individual milk samples. Animals.

[23] Kuhn C., Thaller G., Winter A., Bininda-Emonds O.R.P., Kaupe B., Erhardt G., Bennewitz J., Schwerin M., Fries R. (2004). Evidence for multiple alleles at the *DGAT1* locus better explains a quantitative tip trait locus with major effect on milk fat content in cattle. Genetics.

[24] Buchanan F.C., Fitzsimmons C.J., Van Kessel A.G., Thue T.D., Winkelman-Sim D.C., Schmutz S.M. (2002). Association of a missense mutation in the bovine leptin gene with carcass fat content and leptin mRNA levels. Genet. Sel. Evol..

[25] Roy R., Ordovas L., Zaragoza P., Romero A., Moreno C., Altarriba J., Rodellar C. (2006). Association of polymorphisms in the bovine *FASN* gene with milk-fat content. Anim. Genet..

[26] Littlejohn M.D., Tiplady K., Lopdell T., Law T.A., Scott A., Harland C., Sherlock R., Henty K., Obolonkin V., Lehnert K. (2014). Expression variants of the lipogenic *AGPAT6* gene affect diverse milk composition phenotypes in *Bos taurus*. PLoS ONE.

[27] SAS Institute Inc. (2013). Base SAS 9.4 Procedures Guide: Statistical Procedures.

[28] Rychtářová J., Sztankoova Z., Kyselová J., Zink V., Štípková M., Vacek M., Štolc L. (2014). Effect of *DGAT1*, *BTN1A1*, *OLR1*, and *STAT1* genes on milk production and reproduction traits in the Czech Fleckvieh breed. Czech J. Anim. Sci..

[29] Bouwman A.C., Bovenhuis H., Visker M.H.P.W., van Arendonk J.A.M. (2011). Genome-wide association of milk fatty acids in Dutch dairy cattle. BMC Genet..

[30] Houaga I., Muigai A.W.T., Nganga F.M., Ibeagha-Awemu E.M., Kyallo M., Youssao I.A.K., Stomeo F. (2018). Milk fatty acid variability and association with polymorphisms in *SCD1* and *DGAT1* genes in White Fulani and Borgou cattle breeds. Mol. Biol. Rep..

[31] Nafikov R.A., Schoonmaker J.P., Korn K.T., Noack K., Garrick D.J., Koehler K.J., Minick-Bormann J., Reecy J.M., Spurlock D.E., Beitz D.C. (2014). Polymorphisms in lipogenic genes and milk fatty acid composition in Holstein dairy cattle. Genomics.

[32] Wang X.L., Wurmser C., Pausch H., Jung S., Reinhardt F., Tetens J., Thaller G., Fries R. (2012). Identification and dissection of four major QTL affecting milk fat content in the German Holstein-Friesian population. PLoS ONE.

[33] Bär C., Sutter M., Kopp C., Neuhaus P., Portmann R., Egger L., Reidy B., Bisig W. (2020). Impact of herbage proportion, animal breed, lactation stage and season on the fatty acid and protein composition of milk. Int. Dairy J..

[34] Liu G.F., Yu X., Li S.L., Shao W., Zhang N. (2020). Effects of dietary microalgae (*Schizochytrium* spp.) supplement on milk performance, blood parameters, and milk fatty acid composition in dairy cows. Czech J. Anim. Sci..

[35] Renna M., Ferlay A., Lussiana C., Bany D., Graulet B., Wyss U., Enri S.R., Battaglini L.M., Coppa M. (2020). Relative hierarchy of farming practices affecting the fatty acid composition of permanent grasslands and of the derived bulk milk. Anim. Feed Sci. Tech..

[36] van den Oever S.P., Haselmann A., Schreiner M., Fuerst-Waltl B., Zebeli Q., Mayer H.K., Knaus W. (2021). Hay versus silage: Does hay feeding positively affect milk composition?. Int. Dairy J..

[37] Haselmann A., Wenter M., Fuerst-Waltl B., Zollitsch W., Zebeli Q., Knaus W. (2020). Comparing the effects of silage and hay from similar parent grass forages on organic dairy cows’ feeding behavior, feed intake and performance. Anim. Feed Sci. Tech..

[38] Stergiadis S., Berlitz C.B., Hunt B., Garg S., Givens D.I., Kliem K.E. (2019). An update to the fatty acid profiles of bovine retail milk in the United Kingdom: Implications for nutrition in different age and gender groups. Food Chem..

[39] Młynek K., Danielewicz A., Straczek I. (2021). The effect of energy metabolism up to the peak of lactation on the main fractions of fatty acids in the milk of selected dairy cow breeds. Animals.

[40] Nogalski Z., Wroński M., Sobczuk-Szul M., Mochol M., Pogorzelska P. (2012). The effect of body energy reserve mobilization on the fatty acid profile of milk in high-yielding cows. Asian Australas. J. Anim. Sci..

[41] Stoop W.M., Bovenhuis H., Heck J.M.L., van Arendonk J.A.M. (2009). Effect of lactation stage and energy status on milk fat composition of Holstein-Friesian cows. J. Dairy Sci..

[42] Nantapo C.T.W., Muchenje V., Hugo A. (2014). Atherogenicity index and health-related fatty acids in different stages of lactation from Friesian, Jersey and Friesian x Jersey cross cow milk under a pasture-based dairy system. Food Chem..

[43] Chilliard Y., Ferlay A., Mansbridge R.M., Doreau M. (2000). Ruminant milk fat plasticity: Nutritional control of saturated, polyunsaturated, trans and conjugated fatty acids. Ann. Zootech..

[44] Stergiadis S., Cabeza-Luna I., Mora-Ortiz M., Stewart R.D., Dewhurst R.J., Humphries D.J., Watson M., Roehe R., Auffret M.D. (2021). Unravelling the role of rumen microbial communities, genes, and activities on milk fatty acid profile using a combination of omics approaches. Front. Microbiol..

