# Supplementary material for: Associations among Farm, Breed, Lactation Stage and Parity, Gene Polymorphisms and the Fatty Acid Profile of Milk from Holstein, Simmental and Their Crosses
Source: Animals (Basel). 2021 Nov 17;11(11):3284. doi: 10.3390/ani11113284 (PMC8614357; doi:10.3390/ani11113284)
Supplement: Supplementary file 1 [file animals-11-03284-s001.zip › animals-1410761-supplementary.pdf]

animals

MDPI

Table S1. Sequences of primers used in the polymerase chain reactions and restriction endonucleases used for genotyping.

Locus[1]	Primer	Sequence	Restriction endonuclease
DGAT1	Forward	5′ - GCA CCA TCC TCT TCC TCA AG – 3′	*Cfr*I
	Reverse	5′ - GGA AGC GCT TTC GGA TG – 3′	
LEP	Forward	5′ - ATG CGC TGT GGA CCC CTG TAT C – 3′	*Hph*I
	Reverse	5′ - TGG TGT CAT CCT GGA CCT TCC – 3′	
FASN	Forward	5′ - AGA GCT GAC GGA CTC CAC AC – 3′	*Msc*I
	Reverse	5′ - GCC GAT GCA CTC GAT GTA G – 3′	
SCD1	Forward	5′ - ACC TGG CTG GTG AAT AGT GCT – 3′	*Fnu4H*I
	Reverse	5′ - TCT GGC ACG TAA CCT AAT ACC CT- 3′	
AGPAT6	Forward	5′ - CAA GGC GGC GTA GAC AAA – 3′	Fragment analysis
	Reverse	5′ - AGC CCC GTC AGA GGT TCA T – 3′	

[1] *AGPAT6* = 1-acylglycerol-3-phosphate O-acyltransferase 6, *DGAT1* = acyl-CoA diacylglycerol transferase 1; *LEP* = leptin; *FASN* = fatty acid synthase; *SCD1* = stearoyl CoA desaturase 1.

Table S2. Proportion of fatty acids (FAs) in different genotypes (LSM±SE, *p*-value)[1].

FAs and FA groups[2]			*AGPAT6*			*DGAT1*			*LEP*		*FASN*		*SCD1*		
			CC	*CT*	*TT*	*AA*	*AK*	*MM*	*MW*	*WW*	*AG*	*GG*	*CC*	*CT*	*TT*
C4:0	1	LSM	1.922	1.930	1.975	1.916	1.873	1.914	1.902	1.855	1.897	1.912	1.926	1.886	1.932
		SE	0.070	0.064	0.127	0.066	0.084	0.067	0.073	0.097	0.068	0.066	0.067	0.066	0.076
		p-value		0.883		0.535			0.755		0.658			0.335	
C6:0	2		1.857	1.873	1.898	1.872	1.844	1.863	1.869	1.850	1.862	1.869	1.857	1.857	1.900
			0.045	0.042	0.081	0.044	0.055	0.043	0.047	0.062	0.045	0.043	0.045	0.043	0.050
				0.669		0.528			0.945		0.772			0.343	
C8:0	3		1.386	1.406	1.446	1.405	1.389	1.393	1.415	1.396	1.407	1.398	1.393	1.400	1.437
			0.037	0.034	0.065	0.035	0.044	0.035	0.037	0.050	0.036	0.035	0.036	0.035	0.041
				0.328		0.658			0.542		0.595			0.261	
C10:0	4		3.582	3.665	3.628	3.671	3.634	3.630	3.693	3.683	3.693	3.650	3.583^{Bb}	3.690^{a}	3.828^{A}
			0.115	0.106	0.205	0.110	0.138	0.111	0.119	0.159	0.111	0.108	0.110	0.108	0.126
				0.277		0.736			0.578		0.421			0.010	
C10:1*	5		0.352	0.347	0.373	0.350	0.347	0.349	0.358	0.372	0.352	0.348	0.367^{A}	0.342^{B}	0.312^{C}
			0.015	0.013	0.026	0.014	0.018	0.014	0.015	0.020	0.014	0.014	0.014	0.013	0.015
				0.428		0.834			0.233		0.517			<0.0001	
C11:0	6		0.100	0.103	0.103	0.104	0.101	0.102	0.101	0.114	0.103	0.103	0.100	0.104	0.107
			0.007	0.006	0.012	0.007	0.008	0.007	0.007	0.010	0.007	0.006	0.007	0.007	0.008
				0.744		0.742			0.314		0.955			0.331	
C12:0	7		4.314	4.407	4.355	4.422	4.365	4.364	4.453	4.480	4.470	4.381	4.315^{a}	4.448^{b}	4.574^{b}
			0.144	0.133	0.258	0.137	0.172	0.138	0.147	0.198	0.139	0.134	0.137	0.135	0.157
				0.359		0.680			0.441		0.181			0.028	
C12:1*	8		0.123	0.121	0.127	0.121	0.124	0.122	0.123	0.132	0.124	0.121	0.127^{Aa}	0.121^{Ab}	0.104^{B}
			0.006	0.006	0.012	0.006	0.008	0.006	0.007	0.009	0.005	0.006	0.006	0.006	0.007
				0.608		0.673			0.439		0.311			<0.0001	
C13:0	9		0.144	0.147	0.146	0.148	0.141	0.144	0.145	0.161	0.150	0.145	0.146	0.147	0.151
			0.007	0.007	0.013	0.007	0.008	0.007	0.007	0.010	0.007	0.007	0.007	0.007	0.008
				0.564		0.303			0.115		0.186			0.682	
*iso*C14:0	10		0.141	0.145	0.138	0.146	0.141	0.144	0.151	0.146	0.150^{a}	0.142^{b}	0.145	0.143	0.148
			0.009	0.008	0.015	0.008	0.010	0.009	0.009	0.012	0.008	0.008	0.008	0.008	0.010
				0.560		0.543			0.335		0.045*			0.642	
C14:0	11		12.698	12.764	12.624	12.864	12.635	12.845	12.850	12.610	12.960	12.734	12.642^{Bb}	12.863^{a}	13.198^{Ab}
			0.248	0.228	0.445	0.236	0.297	0.230	0.247	0.331	0.239	0.232	0.237	0.232	0.270
				0.800		0.344			0.699		0.050			0.007	
C14:1n-5*c*	12		1.319	1.287	1.343	1.302	1.306	1.306	1.316	1.413	1.322	1.292	1.393^{A}	1.267^{B}	1.114^{C}
			0.050	0.046	0.088	0.048	0.060	0.050	0.053	0.071	0.049	0.048	0.045	0.044	0.051
				0.287		0.929			0.187		0.201			<0.0001	
*anteiso*C15:0	13		0.476	0.478	0.473	0.485	0.469	0.471	0.481	0.508	0.487	0.477	0.487	0.477	0.475
			0.017	0.016	0.030	0.016	0.020	0.016	0.017	0.023	0.016	0.016	0.016	0.016	0.019
				0.949		0.335			0.100		0.215			0.359	
C15:0	14		1.275	1.285	1.270	1.298	1.241	1.278	1.269	1.376	1.300	1.277	1.290	1.281	1.287
			0.037	0.034	0.066	0.036	0.045	0.035	0.038	0.051	0.036	0.035	0.036	0.036	0.041
				0.814		0.120			0.062		0.179			0.869	
C15:1*	15		0.040	0.040	0.041	0.040	0.040	0.039	0.040	0.043	0.040	0.040	0.040	0.040	0.039
			0.002	0.002	0.003	0.002	0.002	0.002	0.002	0.003	0.002	0.002	0.002	0.002	0.002
				0.500		0.700			0.232		0.522			0.542	
*iso*C16:0	16		0.314	0.322	0.300	0.325	0.312	0.320	0.327	0.336	0.330	0.318	0.323	0.321	0.321
			0.015	0.014	0.027	0.014	0.018	0.015	0.016	0.021	0.015	0.014	0.015	0.014	0.017
				0.369		0.406			0.508		0.074			0.958	
C16:0	17		32.686	32.305	32.774	32.472	32.585	32.596	32.570	32.037	32.353	32.576	32.582	32.400	32.619
			0.543	0.502	0.995	0.524	0.648	0.535	0.569	0.760	0.534	0.517	0.530	0.522	0.604
				0.251		0.827			0.677		0.364			0.657	
C16:1*	18		0.035	0.037	0.043	0.037	0.037	0.036	0.035	0.041	0.037	0.036	0.036	0.037	0.037
			0.002	0.002	0.005	0.002	0.002	0.002	0.002	0.003	0.002	0.002	0.002	0.002	0.002
				0.111		0.867			0.161		0.616			0.219	
C16:1*	19		0.143	0.142	0.143	0.141	0.143	0.140	0.139	0.146	0.140	0.142	0.141	0.142	0.137
			0.005	0.004	0.009	0.004	0.006	0.004	0.005	0.006	0.005	0.004	0.005	0.004	0.005
				0.972		0.555			0.465		0.352			0.364	

FAs and FA groups[2]		*AGPAT6*			*DGAT1*		*LEP*			*FASN*		*SCD1*		
		CC	*CT*	*TT*	*AA*	*AK*	*MM*	*MW*	*WW*	*AG*	*GG*	*CC*	*CT*	*TT*
C16:1n-7*c*	20	1.559	1.526	1.591	1.490	1.565	1.499	1.502	1.606	1.478	1.522	1.447^{Bb}	1.550^{A}	1.561^{a}
		0.070	0.064	0.121	0.067	0.083	0.069	0.073	0.098	0.069	0.066	0.067	0.066	0.076
			0.498		0.261			0.415		0.160			0.002	
*iso*C17:0	21	0.347	0.349	0.347	0.348	0.348	0.346	0.345	0.359	0.348	0.348	0.351^{A}	0.349^{A}	0.336^{B}
		0.007	0.007	0.013	0.007	0.009	0.007	0.007	0.010	0.007	0.007	0.007	0.007	0.008
			0.803		0.955			0.263		0.999			0.013	
C16:1*	22	0.193	0.193	0.200	0.194	0.194	0.193	0.199	0.204	0.199^{a}	0.192^{b}	0.198^{A}	0.193^{a}	0.183^{Bb}
		0.007	0.007	0.013	0.007	0.009	0.007	0.008	0.010	0.007	0.007	0.007	0.007	0.008
			0.845		0.941			0.163		0.042			0.026	
*anteiso*C17:0	23	0.432	0.434	0.418	0.435	0.427	0.428^{B}	0.431^{b}	0.458^{Aa}	0.435	0.432	0.435	0.433	0.429
		0.009	0.008	0.016	0.009	0.011	0.009	0.009	0.012	0.009	0.008	0.009	0.008	0.010
			0.490		0.335			0.011		0.536			0.639	
C17:0	24	0.543	0.547	0.542	0.544	0.549	0.542	0.529	0.574	0.548	0.544	0.549	0.546	0.530
		0.018	0.016	0.028	0.017	0.021	0.017	0.019	0.025	0.017	0.016	0.017	0.017	0.019
			0.900		0.777			0.124		0.624			0.325	
C17:1n-7*c*	25	0.293	0.293	0.271	0.285	0.307	0.288	0.283	0.315	0.289	0.290	0.284	0.295	0.289
		0.015	0.014	0.028	0.015	0.018	0.014	0.015	0.021	0.015	0.014	0.015	0.014	0.017
			0.665		0.152			0.234		0.827			0.307	
C18:0	26	8.202	8.300	7.679	8.325	8.099	8.257	8.204	8.211	8.271	8.272	8.414	8.218	8.010
		0.316	0.291	0.559	0.301	0.376	0.305	0.325	0.436	0.307	0.297	0.304	0.299	0.348
			0.387		0.458			0.950		0.996			0.176	
C18:1*t**	27	0.384	0.383	0.395	0.384	0.366	0.377	0.377	0.374	0.381	0.380	3.842	0.379	0.371
		0.012	0.011	0.021	0.011	0.014	0.012	0.013	0.017	0.012	0.011	0.011	0.011	0.013
			0.822		0.105			0.976		0.856			0.275	
C18:1n-7*t*	28	1.317	1.360	1.385	1.365	1.327	1.363	1.325	1.359	1.380	1.343	1.371	1.351	1.336
		0.046	0.042	0.081	0.043	0.054	0.045	0.048	0.065	0.044	0.043	0.044	0.043	0.050
			0.088		0.380			0.336		0.075			0.497	
C18:1n-9*c*	29	18.020	18.006	17.928	17.743	18.367	17.940	17.910	17.923	17.707	17.969	17.871	17.959	17.574
		0.525	0.483	0.941	0.494	0.622	0.490	0.525	0.704	0.502	0.487	0.501	0.492	0.573
			0.993		0.217			0.994		0.279			0.533	
C18:1n-7*c*	30	0.692	0.694	0.659	0.685	0.701	0.689	0.672	0.688	0.677	0.695	0.687	0.696	0.665
		0.029	0.026	0.052	0.027	0.034	0.026	0.028	0.038	0.028	0.027	0.027	0.027	0.031
			0.757		0.570			0.554		0.186			0.257	
C18:1*	31	0.379	0.382	0.385	0.382	0.375	0.379	0.375	0.387	0.385	0.378	0.385	0.377	0.379
		0.011	0.010	0.018	0.010	0.013	0.011	0.011	0.015	0.010	0.010	0.010	0.010	0.012
			0.825		0.483			0.565		0.136			0.179	
C18:1*	32	0.367	0.368	0.366	0.370	0.363	0.371	0.364	0.364	0.373	0.365	0.375^{a}	0.364^{b}	0.368^{b}
		0.010	0.009	0.018	0.010	0.012	0.010	0.011	0.014	0.010	0.010	0.010	0.010	0.008
			0.923		0.549			0.408		0.082			0.018	
C18:2*	33	0.139	0.138	0.145	0.136	0.141	0.137	0.136	0.143	0.137	0.137	0.132^{B}	0.140^{A}	0.146^{A}
		0.006	0.005	0.010	0.005	0.007	0.005	0.006	0.008	0.006	0.005	0.005	0.005	0.006
			0.662		0.339			0.583		0.971			0.0006	
C18:2n-6**	34	2.094	2.104	2.172	2.097	2.105	2.091	2.059	2.142	2.098	2.099	2.121	2.083	2.094
		0.054	0.050	0.095	0.051	0.064	0.051	0.054	0.073	0.053	0.051	0.052	0.051	0.060
			0.640		0.866			0.358		0.987			0.284	
C19:1*	35	0.137	0.138	0.140	0.138	0.137	0.137	0.137	0.151	0.138	0.137	0.139	0.137	0.133
		0.005	0.005	0.009	0.005	0.006	0.005	0.005	0.007	0.005	0.005	0.005	0.005	0.005
			0.911		0.892			0.063		0.521			0.242	
C18:3*	36	0.058	0.057	0.058	0.058	0.057	0.056^{B}	0.056^{B}	0.067^{A}	0.057	0.058	0.058	0.058	0.057
		0.002	0.002	0.004	0.002	0.003	0.002	0.002	0.003	0.002	0.002	0.002	0.002	0.002
			0.732		0.646			0.0002		0.681			0.829	
C18:3n-3**	37	0.398	0.377	0.376	0.374	0.376	0.377	0.363	0.388	0.379	0.371	0.378	0.372	0.373
		0.013	0.012	0.024	0.013	0.016	0.013	0.014	0.018	0.013	0.013	0.013	0.013	0.015
			0.334		0.858			0.125		0.238			0.554	
C20:0	38	0.111	0.114	0.105	0.115	0.110	0.112	0.113	0.117	0.115	0.113	0.114	0.114	0.113
		0.004	0.004	0.007	0.004	0.005	0.004	0.004	0.005	0.004	0.004	0.004	0.004	0.004
			0.138		0.220			0.598		0.314			0.959	

FAs and FA groups[2]		*AGPAT6*			*DGAT1*		*LEP*			*FASN*		*SCD1*		
		CC	*CT*	*TT*	*AA*	*AK*	*MM*	*MW*	*WW*	*AG*	*GG*	*CC*	*CT*	*TT*
C18:2*c*9*t*11	39	0.436	0.445	0.473	0.445	0.441	0.449	0.440	0.460	0.452	0.439	0.439	0.448	0.448
		0.018	0.017	0.032	0.018	0.022	0.018	0.019	0.026	0.018	0.017	0.018	0.017	0.020
			0.333		0.840			0.572		0.139			0.585	
C20:1n-9*c*	40	0.108	0.109	0.117	0.108	0.108	0.108	0.109	0.116	0.111^{a}	0.107^{b}	0.110	0.108	0.106
		0.004	0.004	0.008	0.004	0.005	0.004	0.005	0.006	0.004	0.004	0.004	0.004	0.005
			0.505		0.937			0.375		0.046			0.499	
C20:1n-7*c*	41	0.047	0.049	0.049	0.048	0.051	0.048	0.048	0.049	0.049	0.049	0.048	0.049	0.049
		0.002	0.002	0.005	0.002	0.003	0.002	0.002	0.003	0.002	0.002	0.002	0.002	0.003
			0.274		0.162			0.834		0.993			0.789	
C21:0	42	0.026	0.025	0.023	0.025	0.026	0.024	0.025	0.026	0.025	0.025	0.025	0.025	0.026
		0.001	0.002	0.004	0.001	0.002	0.001	0.002	0.002	0.001	0.001	0.001	0.001	0.002
			0.699		0.547			0.500		0.405			0.634	
C20:3n-6**	43	0.073	0.074	0.081	0.073	0.077	0.073	0.075	0.080	0.075	0.073	0.076	0.073	0.071
		0.004	0.003	0.007	0.004	0.004	0.004	0.004	0.005	0.004	0.004	0.004	0.004	0.004
			0.466		0.207			0.326		0.321			0.157	
C20:4n-6**	44	0.108	0.111	0.120	0.109	0.109	0.106^{B}	0.109^{b}	0.120^{Aa}	0.109	0.109	0.111	0.108	0.108
		0.004	0.004	0.007	0.004	0.005	0.004	0.004	0.006	0.004	0.004	0.004	0.004	0.005
			0.095		0.904			0.006		0.764			0.285	
C22:0	45	0.041	0.042	0.041	0.043^{a}	0.039^{b}	0.042	0.042	0.046	0.043	0.042	0.043	0.042	0.041
		0.002	0.002	0.004	0.002	0.003	0.002	0.002	0.003	0.002	0.002	0.002	0.002	0.002
			0.361		0.030			0.267		0.531			0.497	
C20:4n-3**	46	0.030	0.031	0.034	0.029^{B}	0.035^{A}	0.030	0.030	0.030	0.032	0.030	0.031	0.030	0.029
		0.002	0.002	0.004	0.002	0.003	0.002	0.002	0.003	0.002	0.002	0.002	0.002	0.002
			0.461		0.003			0.945		0.066			0.475	
C20:5n-3**	47	0.031	0.031	0.037	0.031	0.032	0.030^{b}	0.030^{b}	0.036^{a}	0.031	0.032	0.031	0.031	0.031
		0.002	0.002	0.004	0.002	0.003	0.002	0.002	0.003	0.002	0.002	0.002	0.002	0.002
			0.180		0.636			0.042		0.515			0.972	
C24:0	48	0.042	0.045	0.043	0.044	0.041	0.043	0.043	0.046	0.044	0.044	0.044	0.044	0.043
		0.002	0.002	0.004	0.002	0.003	0.002	0.002	0.003	0.002	0.002	0.002	0.002	0.002
			0.096		0.159			0.509		0.919			0.590	
C22:5n-3**	49	0.059	0.062	0.062	0.054	0.051	0.053^{B}	0.053^{B}	0.063^{A}	0.056^{a}	0.053^{b}	0.054	0.054	0.053
		0.002	0.002	0.005	0.003	0.004	0.004	0.004	0.004	0.004	0.003	0.004	0.003	0.004
			0.139		0.288			0.005		0.021*			0.718	
SFA	50	68.958	68.951	68.765	69.248	68.603	69.096	69.186	68.545	69.212	69.057	69.001	69.042	69.706
		0.639	0.588	1.145	0.060	0.758	0.597	0.640	0.857	0.612	0.593	0.609	0.598	0.697
			0.983		0.296			0.710		0.599			0.268	
MUFA	51	25.513	25.473	25.563	25.185	25.859	25.381	25.305	25.692	25.186	25.406	25.400	25.414	24.754
		0.587	0.540	1.052	0.552	0.696	0.547	0.587	0.786	0.561	0.544	0.559	0.549	0.639
			0.985		0.234			0.862		0.416			0.227	
TFA	52	2.135	2.188	2.256	2.192	2.135	2.187	2.141	2.194	2.210	2.161	2.190	2.177	2.153
		0.067	0.062	0.119	0.064	0.080	0.067	0.071	0.095	0.065	0.063	0.065	0.064	0.074
			0.148		0.371			0.469		0.109			0.754	
PUFA	53	3.339	3.369	3.499	3.351	3.372	3.343	3.300	3.475	3.370	3.347	3.374	3.343	3.355
		0.080	0.073	0.141	0.076	0.095	0.076	0.081	0.108	0.077	0.075	0.077	0.076	0.088
			0.375		0.788			0.185		0.537			0.680	
PUFAn-3	54	0.431	0.441	0.448	0.438	0.442	0.438	0.427	0.476	0.445	0.435	0.444	0.437	0.018
		0.016	0.015	0.029	0.015	0.019	0.015	0.017	0.022	0.016	0.015	0.016	0.015	
			0.375		0.808			0.059		0.210			0.512	
SCFA	55	13.940	14.162	14.257	14.166	13.975	14.037	14.213	14.190	14.213	14.078	13.982	14.157	14.465
		0.355	0.327	0.634	0.340	0.427	0.340	0.363	0.485	0.345	0.335	0.342	0.336	0.392
			0.354		0.582			0.631		0.412			0.178	
MCFA	56	52.558	52.176	52.677	52.419	52.306	52.475	52.497	52.221	52.425	52.384	53.357	52.355	52.698
		0.636	0.585	1.300	0.607	0.759	0.612	0.657	0.882	0.618	0.600	0.616	0.606	0.705
			0.373		0.853			0.940		0.888			0.714	
LCFA	57	33.448	33.637	33.037	33.390	33.744	33.495	33.270	33.573	33.336	33.516	33.653	33.461	32.811
		0.806	0.741	1.450	0.759	0.957	0.760	0.816	1.095	0.771	0.747	0.768	0.754	0.877
			0.800		0.649			0.870		0.627			0.354	

**FAs and FA groups**[2]		*AGPAT6*			*DGAT1*		*LEP*			*FASN*		*SCD1*		
		CC	***CT***	***TT***	***AA***	***AK***	***MM***	***MW***	***WW***	***AG***	***GG***	***CC***	***CT***	***TT***
UFA	58	28.849	28.838	29.058	28.535	29.215	28.715	28.597	29.166	28.554	28.751	28.773	28.755	28.104
		0.626	0.575	1.120	0.589	0.742	0.583	0.624	0.836	0.599	0.581	0.596	0.586	0.682
			0.976		0.260			0.752		0.495			0.273	
BCFA	59	1.854	1.871	1.826	1.882	1.838	1.851^{b}	1.884	1.958^{a}	1.894^{a}	1.859^{b}	1.886	1.865	1.845
		0.040	0.036	0.070	0.037	0.047	0.038	0.041	0.054	0.038	0.037	0.038	0.037	0.043
			0.492		0.241			0.029		0.048			0.300	
C18	60	28.628	28.749	28.096	28.511	28.925	28.628	28.481	28.611	28.430	28.676	28.768	28.594	27.998
		0.736	0.677	1.322	0.692	0.973	0.689	0.740	0.994	0.704		0.768	0.688	0.8001
			0.816		0.561			0.934		0.469			0.352	

[a,b] different letters between genotypes in the same row represent significant differences at $p < 0.05$.
[A,B] different letters between genotypes in the same row represent significant differences at $p < 0.01$.
[1] LSM = Least Square Mean; SE = Standard Error; *p*-value = *p*-value of the polymorphism effect; $p < 0.05$ (red highlighted) $p < 0.10$ (green highlighted).
[2] FA proportions were stated as g/100 g of FAs; * = unidentified position isomer; ** = all-*cis* isomer; *c* = *cis* isomer; *t* = *trans* isomer; SFA = saturated FA; MUFA = monounsaturated FA; TFA = *trans* isomers of unsaturated FA; PUFA = polyunsaturated FA; PUFAn-3 = the sum of polyunsaturated FA n-3; SCFA = short-chain FA; MCFA = medium-chain FA; LCFA = long-chain FA; UFA = unsaturated FA; BCFA = branched-chain FA; C18 = the sum of FA with C18.
